# Artificial intelligence applications in pediatric oncology diagnosis

**DOI:** 10.37349/etat.2023.00127

**Published:** 2023-02-28

**Authors:** Yuhan Yang, Yimao Zhang, Yuan Li

**Affiliations:** 1 Department of Pediatric Surgery, West China Hospital, Sichuan University, Chengdu 610041, Sichuan, China; 2 Laboratory of Digestive Surgery, State Key Laboratory of Biotherapy and Cancer Center, Department of Pediatric Surgery, West China Hospital, Sichuan University, Chengdu 610041, Sichuan, China; University of Campania “L. Vanvitelli”, Italy

**Keywords:** Pediatric oncology, artificial intelligence, cancer diagnosis, machine learning, deep learning

## Abstract

Artificial intelligence (AI) algorithms have been applied in abundant medical tasks with high accuracy and efficiency. Physicians can improve their diagnostic efficiency with the assistance of AI techniques for improving the subsequent personalized treatment and surveillance. AI algorithms fundamentally capture data, identify underlying patterns, achieve preset endpoints, and provide decisions and predictions about real-world events with working principles of machine learning and deep learning. AI algorithms with sufficient graphic processing unit power have been demonstrated to provide timely diagnostic references based on preliminary training of large amounts of clinical and imaging data. The sample size issue is an inevitable challenge for pediatric oncology considering its low morbidity and individual heterogeneity. However, this problem may be solved in the near future considering the exponential advancements of AI algorithms technically to decrease the dependence of AI operation on the amount of data sets and the efficiency of computing power. For instance, it could be a feasible solution by shifting convolutional neural networks (CNNs) from adults and sharing CNN algorithms across multiple institutions besides original data. The present review provides important insights into emerging AI applications for the diagnosis of pediatric oncology by systematically overviewing of up-to-date literature.

## Introduction

The exponentially growing knowledge and techniques have explored innovative perspectives for multi-layered diagnoses in pediatric oncology that the expectation of patients and their families have been developed for their specific situation to receive optimized care instantly and comprehensively. Artificial intelligence (AI) strategies can tackle enormous amounts of original data in a short time to solve complex tasks with high accuracy [1, 2]. Physicians can improve their diagnostic efficiency with the assistance of AI techniques for improving the subsequent personalized treatment and surveillance.

AI has developed into various computer-assisted theories and is mainly implemented with working principles of machine learning (ML) and deep learning (DL). AI algorithms fundamentally capture data, identify underlying patterns, achieve preset endpoints, and provide decisions and predictions about real-world events. ML, as a main subset of AI, indicates a different flowchart compared with traditional hard-coded software programs which apply algorithms to construct predictive models dynamically by training large amounts of historical data. DL, as a growing aspect of AI, represents benefits in learnable weights and high efficiency with minimal pre-processing based on the structure of convolutional neural networks (CNNs) with multiple inter-connected layers. DL-CNNs composed of multiple stacked CNN layers have advantages in accurate, faster, vendor-independent processing compared with ML algorithms applied previously [3]. Notably, AI methods have been demonstrated to offer effective assistance for clinical management, including cancer segmentation, susceptibility, and classification, as essential fundamental for early diagnosis and prognosis management in cancer research [4, 5].

The application of AI not only makes full use of the various aspects of clinical diversity but also helps to address the current lack of objectivity and universality in expert systems [6]. The application of AI can help hospitals train junior physicians in clinical diagnosis and decision-making. A growing number of research papers are reporting the impressive diagnostic performance of computer systems built using ML [7]. DL techniques, in particular, are transforming their ability to interpret imaging data [8, 9]. These results may improve sensitivity and ensure fewer false positives than radiologists. However, DL techniques also run the risk of overfitting the training data, resulting in a brittle degraded performance in certain settings [10]. Thus, AI often involves a tradeoff between accuracy and intelligibility.

Despite the current AI advancements, the sustainable development of health AI tools relies on the availability of large datasets with strict quality control [11]. Several biomedical imaging repositories have been created to date [12, 13], such as The Cancer Imaging Archive (TCIA) one of the most renowned repositories focusing on cancer imaging [14]. Nowadays, there have been specific data repositories for pediatric cancer, for example, the PRIMAGE project, as an open cloud-based platform based on European populations, involves high-quality multidimensional anonymized datasets (imaging, clinical, molecular, and genetics) for the training and validation of ML and multiscale algorithms [15]. Albeit of huge potential, the vast majority of these repositories have been created as stand-alone entities, being currently not in a position to become interoperable with similar existing initiatives. As such, the creation of a fully findable, accessible, interoperable, reusable (FAIR) repository based on multiple populations is still warranted for AI analysis [16].

The prosperous development of AI techniques promotes numerous potential applications in pediatric oncology with two main bottlenecks for successful utilities, including the need for large data entry and a strong graphic processing unit (GPU) with appropriate computer and memory power. AI algorithms provide timely references based on large amounts of clinical and imaging data and sufficient GPU power [17]. Meanwhile, DL-CNNs can learn from medical literature automatically to capture innovative and feasible ideas for literature reviews, which can assist in accurate diagnosis at an early stage and optimal treatment selection for individuals [18]. Nowadays, it’s necessary to feed AI algorithms with representative large-sample data sets, hundreds or thousands for typical cases where sparse and/or unqualified data induces unsatisfactory performance and unreliable outcomes [18]. Currently, neither ML nor DL can guarantee accuracy and consistency in specific circumstances without similarity compared with historical training data. This is an inevitable challenge especially for pediatric oncology because of the low morbidity and individual heterogeneity. However, this problem may be solved in the near future considering the exponential advancements of AI algorithms technically to decrease the dependence of AI operation on the amount of data sets and the efficiency of computing power. For instance, it could be a feasible solution by shifting CNNs from adults and sharing CNN algorithms across multiple institutions besides original data. The present review provides important insights into emerging AI applications for the diagnosis of pediatric oncology by systematically overviewing updated literature.

## Current AI applications in the diagnosis of pediatric oncology

Clinicians make clinical diagnoses depending on their professional knowledge and clinical experience through signs, symptoms, laboratory, and imaging examinations. It seems hard to guarantee diagnostic accuracy and consistency by dealing with abundant and multidimensional data from the human brain. The AI algorithms have advantages in learning and training vast amounts of data and integrating it into a certain outcome in a very short time, which allows efficiency and effectiveness of computer-aided diagnosis (CAD) in clinical practices. DL algorithms have recently made significant progress in extracting and processing information from medical images, which have been applied in various medical tasks extensively not only in radiology and pathology with satisfactory performance comparable to or even superior to that of human experts. Notably, DL algorithms could identify underlying information from medical images associated with tumor diagnosis [19]. The AI applications in the diagnosis of pediatric oncology are summarized in specific cancer types using ML and/or DL methods.

### Non-solid tumor diagnosis

A performance comparison of diagnosis in pediatric hematological malignancies using AI strategies is shown in Table 1. The results of cluster and discriminant analyses for various types of pediatric acute leukemia revealed that a combination of DL analysis and microscopic blood images facilitated the classification of acute leukemia and outperformed expert hematologists with an accuracy of more than 98% [20, 21]. The utility of AI in the automatic analysis of microscopy images represented diagnostic accuracy of around 95% in acute promyelocytic leukemia [22], acute lymphoblastic leukemia (ALL) [23, 24], and leukemic B-lymphoblast [25], which was optimized by a hybrid model using a genetic algorithm and a residual CNN reaching an accuracy of 98.46% [26]. The DL analysis was also applied in the classification of ALL, acute myeloid leukemia (AML), and chronic myeloid leukemia (CML) using bone marrow cell microscopy images [27]. Otherwise, an easy-to-interpret transcriptome-wide biomarker was developed for accurate ALL subtyping by elucidating diagnostic associations between messenger RNA (mRNA) sequencing profiles and ALL lesions [28]. The ML-based strategies on DNA methylation showed advantages in the differentiation of leukemia blood from normal blood [29, 30].

**Table 1.** Current AI applications in the diagnosis of childhood non-solid tumor

**AI method**	**Medical field**	**Task**	**Tumor**	**Result**
CNN/GAN	Pathology	Detecting ALL and AML using a deep learner classifier using microscopic blood images	ALL and AML	ACC: 98%–98.67%	[20, 21]
CNN and GAN	Pathology/Genomics	Constructing a hybrid model using a genetic algorithm and a residual CNN to predict ALL using microscopy images	ALL	ACC: 98.46%	[26]
SVM	Pathology	Building a model to classify acute leukemias using flow cytometry	Acute promyelocytic leukemia	ACC: 94.2%; AUC: 99.5	[22]
ANN/FFNN/SVM	Pathology	Proposing a ML-based model for ALL categorization using microscopic blood images	ALL	ACC: 98.1–100%	[23, 24]
CNN	Pathology	Building an aggregated DL model for leukemic B-lymphoblast classification	Leukemic B-lymphoblast	ACC: 96.58%	[25]
CNN	Pathology	Using bone marrow cell microscopy images for the classification of AML, ALL, and CML	AML, ALL, and CML	ACC: 90–99%	[27]
RF	Others-mRNA sequencing	Developing transcriptome-wide biomarkers for ALL subtyping	ALL	ACC: 97–100%	[28]
ANN	Others-DNA methylation	Identifying reliable cancer-associated methylation signals in gene regions from leukemia patients	Leukemia	ACC: 93.8%	[29]
Nearest shrunken centroids	Others-DNA methylation	Investigating the utility of CpG methylation status to differentiate blood from patients with ALL and AML from normal blood	ALL and AML	AUC: 99.98	[30]

GAN: generative adversarial network; SVM: support vector machine; ANN: artificial neural network; FFNN: feed forward neural network; ACC: accuracy; AUC: area under the curve; RF: random forest; CpG: cytosine-guanine

### Solid tumor diagnosis

#### Intracranial tumor diagnosis

A performance comparison of diagnosis in pediatric intracranial tumors using AI strategies is shown in Table 2. Recently, the use of an ML-based classification model has been shown to improve the diagnostic accuracy of childhood intracranial tumors, especially for posterior fossa tumors by analyzing multiple magnetic resonance imaging (MRI) sequences that the DL-CNN model showed improved accuracy with similar sensitivity and improved specificity in terms of discriminate several hard-to-differentiate brain tumor subtypes compared to radiologists’ recognition [2]. The improved performance of these AI-based models warranted further verification in prospective studies and even randomized clinical trials for broad clinical practice. For different tumor subgroups coupled with overlapping neuroimaging features, the use of DL-CNNs could identify these atypical cases with high discriminative performance (AUC: 0.81–0.98), which provided low-cost and high-efficiency decision-making support for reference of diagnostic brain biopsy or maximal tumor resection [31–40]. Moreover, a novel gene-derived algorithm improved the performance of AI-based models with small-scale data by identifying optimal architectures using feature embeddings from state-of-the-art image classification networks that this approach might offer an available solution for the small-sample task in pediatric brain tumors [41].

**Table 2.** Current AI applications in the diagnosis of childhood intracranial tumor

**AI method**	**Medical field**	**Task**	**Tumor**	**Result**	**References**
SVM	Radiology	Classifying pediatric posterior fossa tumors	Pediatric posterior fossa tumors	ACC: 75–85%	[32]
LR/XGB/LASSO	Radiology	Distinguishing pediatric supratentorial tumors, high-grade gliomas, and ependymomas	Supratentorial embryonal tumors, high-grade gliomas, and ependymomas	ACC: 81–91%; AUC: 0.82–0.98	[33]
CNN	Radiology	Establishing a pre-trained ResNet18 with transfer learning to identify germinomas of the basal ganglia	Gliomas and germinomas	AUC: 0.88	[34]
CNN	Radiology	Training on T1-weighted gadolinium-enhanced MRI scans of glioblastomas, atypical primary central nervous system lymphomas, and solitary brain metastasis	Glioblastomas, atypical primary central nervous system lymphomas, and solitary brain metastasis	AUC: 0.81–0.98	[31]
CNN/RF/DT/KNN/SVM	Radiology	Classifying primary central nervous system lymphoma and glioma types	Primary central nervous system lymphoma and glioma	ACC: 84%; AUC: 0.839	[35]
LR/ANN	Radiology	Developing a sequential ML classifier to distinguish medulloblastoma from ependymoma	Medulloblastoma and ependymoma	ACC: 94–95.5%	[36]
SVM/LR/KNN/RF/XGB/ANN	Radiology	Distinguishing atypical teratoid/rhabdoid tumors and medulloblastomas by MR imaging-based radiomic phenotypes	Atypical teratoid/rhabdoid tumors and medulloblastomas	ACC: 81%; AUC: 0.86	[37]
CNN	Radiology	Characterizing and classifying multiple tumor histologic features in pediatric high-grade brain tumors employing diffusion basis spectrum imaging	Pediatric high-grade brain tumors	AUC: 0.950–0.991	[38]
GBDT	Radiology	Applying multiparametric MRI to differentiate pilocytic astrocytoma from cystic oligodendrogliomas	Pilocytic astrocytoma and cystic oligodendrogliomas	AUC: 0.99	[39]
CNN	Radiology	Developing an MR imaging-based DL model for posterior fossa tumor detection and tumor pathology classification	Diffuse midline glioma of the pons, medulloblastoma, pilocytic astrocytoma, and ependymoma	ACC: 92%; AUC: 0.99	[40]
CNN	Radiology	Identifying the pediatric brain tumor, adamantinomatous craniopharyngioma	Adamantinomatous craniopharyngioma	ACC: 83.3–87.8%	[41]
CNN	Pathology	Proposing a time-efficient and reliable CAD for the automatic diagnosis of pediatric medulloblastoma and its subtypes from histopathological images	Medulloblastoma	ACC: 90–100%	[42, 43]
SVM	Others-blood markers	Differentiating malignant and benign pediatric brain tumors using blood markers	Pediatric brain tumors	ACC: 71.6%	[45]
LR	Others-Raman spectroscopy	Investigating the potential for Raman spectroscopy to accurately diagnose pediatric brain tumors intraoperatively	Pediatric brain tumors	AUC: 0.91–0.94	[46]
RF	Others-DNA methylation profiles	Describing a fast and cost-efficient workflow for intraoperative classification of brain tumors based on DNA methylation profiles generated by low coverage nanopore sequencing and ML algorithms	Pediatric brain tumors	ACC: 89%	[47]
SVM	Others-proteomics of cerebrospinal fluid	Distinguishing among brain tumor *versus* non-tumor/hemorrhagic conditions and differentiating two large classes of brain tumors	Pediatric brain tumors	AUC: 0.97–1	[48]
LASSO	Others-lncRNAs	Developing an RF-based ML algorithm identifying a lncRNA-based diagnostic signature	Medulloblastoma	AUC: 0.974–1	[44]

LR: logistic regression; XGB: extreme gradient boosting; LASSO: least absolute shrinkage and selection operator; ResNet18: residual neural network with 18-layer by using more 5-layer blocks; DT: decision tree; KNN: k-nearest neighbour; GBDT: gradient boosting decision tree; lncRNAs: long non-coding RNAs

Attallah [42, 43] proposed two time-efficient and reliable CAD systems called MB-AI-His and CoMB-Deep for the automatic diagnosis of pediatric medulloblastoma and its subtypes based on histopathological whole-slide images. These systems combined the benefits of DL techniques and textural analysis feature extraction methods through a cascaded manner to achieve diagnostic and time efficiency simultaneously. The subtypes of pediatric medulloblastoma could also be accurately identified by an RF-based ML algorithm based on 11 lncRNA variables [44]. Otherwise, some innovative techniques were applied to diagnose pediatric brain tumors using AI techniques intraoperatively. Khayat Kashani et al. [45] used inflammatory indicators on peripheral blood tests to classify pediatric benign and malignant brain tumors. Jabarkheel et al. [46] explored the intraoperative diagnostic potential of Raman spectroscopy using an ML classifier in pediatric brain tumors. This ML-based method differentiated the normal brain from neoplastic tissue in a non-invasive manner and efficient diagnostic performance (AUC > 0.90) compared with microscopic visualization and intraoperative navigation. Djirackor et al. [47] applied DNA methylation nanopore sequencing and ML algorithms for the intraoperative classification of brain tumors. This approach performed correct diagnosis in all six cases with a median operating time of 97 min, which assisted decision-making for surgeons within a timeframe. Bruschi et al. [48] used waste cerebrospinal fluid from extraventricular drainage and used proteome analysis to distinguish patients with brain tumors *versus* non-tumor/hemorrhagic and classify subtypes of brain tumors.

#### Extracranial tumor diagnosis

AI techniques have been applied in the diagnosis of pediatric extracranial tumors, mainly including soft-tissue and bone tumors (Table 3). For bone tumors, AI-based algorithms were used to classify benign and malignant lesions based on multiple radiological images, such as X-ray and MRI [49]. He et al. [50] and Pan et al. [51] proposed DL-CNN models with similar accuracy compared to subspecialists and better performance than junior radiologists. The same strategy was applied in pediatric soft-tissue sarcoma a ML-based model yielded great discriminative performance for differentiation between malignant and benign soft-tissue masses with an accuracy of 90.5%, sensitivity of 100%, and specificity of 80.6% [52] as well as other types of sarcomas *versus* benign diseases [53, 54]. The AI-based techniques on radiological images have been only applied in extremity tumors and the diagnostic potential of AI-based strategies needs further exploration in other tumors, such as abdominal and chest tumors.

**Table 3.** Current AI applications in the diagnosis of childhood extracranial tumor

**AI method**	**Medical field**	**Task**	**Tumor**	**Result**	**References**
CNN	Radiology	Developing an AI algorithm to distinguish osteomyelitis from Ewing sarcoma	Ewing sarcoma and osteomyelitis	ACC: 86.7–94.4%	[53]
CNN/SVM	Radiology	Constructing image-based models to identify well-differentiated liposarcoma and lipoma	Well-differentiated liposarcomas and lipomas	ACC: 86.84%; AUC: 0.942	[54]
CNN/RF	Radiology	Developing a DL/ML model to classify primary bone tumors from preoperative radiographs and compare performance with radiologists	Malignant and benign bone tumors	AUC: 0.79–0.97	[49–51]
SVM/GLM/RF	Radiology	Constructing a radiomics-based machine method for differentiation between malignant and benign soft-tissue masses	Malignant and benign soft-tissue masses	AUC: 0.88–0.96; ACC: 80.8–90.5%	[52]
CNN	Pathology	Building CNNs for rhabdomyosarcoma histology subtype classification	Rhabdomyosarcoma	AUC: 0.92–0.94	[55]
CNN	Pathology	Developing a DL CNN-based differential diagnosis system on soft-tissue sarcoma subtypes based on whole histopathology tissue slides	Soft-tissue sarcoma	AUC: 0.889	[56]
LDA	Pathology	Identifying proteomic differences, which would more reliably differentiate between benign and malignant melanocytic lesions	Benign nevi and melanomas	SEN: 98.76%; SPE: 99.65%	[59]
CNN	Others-dermatological photos	Establishing an AI algorithm to diagnose infantile hemangiomas based on clinical images	Infantile hemangiomas	ACC: 91.7%	[61]
LR	Others-umbilical cord blood sera	Exploring prediction biomarkers for infantile hemangiomas using noninvasive umbilical cord blood	Infantile hemangiomas	AUC: 0.756–0.943	[62]
CNN	Others-dermoscopic examination	Developing a DCNN model to support dermatologists in the classification and management of atypical melanocytic skin lesions	Early melanomas and atypical nevi	AUC: 0.903	[60]
SVM	Others-cell-free DNA	Providing a comprehensive analysis of circulating tumor DNA beyond recurrent genetic aberrations for early diagnosis	Ewing sarcoma and other pediatric sarcomas	SEN: 73%; SPE: 100%	[57]
SVM	Others-electronic colorimeters	Determining the diagnostic utility of widely available colorimetric technology when differentiating port-wine birthmarks from infantile hemangiomas in photographs of infants less than 3 months old	Port-wine birthmarks and infantile hemangiomas	ACC: 90%	[63]
DT & RF	Others-array-generated DNA methylation data	Classifying soft tissue and bone tumors using an ML classifier algorithm based on array-generated DNA methylation data	Soft tissue and bone tumors	AUC: 0.999	[58]

GLM: general linear model; LDA: linear discriminant analysis; DCNN: deep CNN

Zhang et al. [55] and Frankel et al. [56] developed DL-CNN differential diagnosis system with an AUC of 0.889 for pre-pathologist screening and quantifying diagnosis likelihood of trained soft-tissue sarcoma subtypes based on whole histopathology tissue slides. Considering the limited worldwide availability of sarcoma pathology expertise, this AI-based approach suggested assistance for local pathologists to quickly narrow the differential diagnosis of the sarcoma subtype in children, adolescents, and young adults. The cell-free DNA of Ewing sarcoma and other pediatric sarcomas for liquid biopsy could achieve sensitive detection and classification in peripheral blood independent of any genetic alterations [57]. The potential of array-generated DNA methylation data in early-stage diagnosis and classification was explored based on an ML-based classifier algorithm [58]. Otherwise, the CAD strategy was applied in the detection and diagnosis of childhood dermatological tumors using histopathological and dermoscopic data. Lazova et al. [59] performed ML-based histopathology-guided mass spectrometry profiling analysis on formalin-fixed, paraffin-embedded tissue samples to identify the proteomic level and achieve a sensitivity of 98.76% and specificity of 99.65% in determining benign nevi and malignant melanomas. Tognetti et al. [60] applied dermoscopic images to develop a DL-CNN model in the classification between atypical nevi from early melanomas, which achieved adequate accuracy (AUC: 90.3, sensitivity: 86.5%, and specificity: 73.6%) and eliminated the influence from dermatologists’ experience. Several AI-based algorithms on novel resources also achieved accurate diagnostic performance for skin neoplasms including photos of skin neoplasms [61], umbilical cord blood sera [62], and electronic colorimeters [63].

### AI applications in the identification of diagnostic signature

AI-based principles have been used for the detection and segmentation of pediatric malignant tumors. For example, Wu et al. [64] used a residual fusion network to detect osteosarcomas on MRI scans; Peng et al. [65] used a CNN for automated pediatric brain tumor detection and segmentation on MRI scans with automatic two-dimensional (2D) and volumetric size measurement of tumors; Strijbis et al. [66] used a CNN for automated eye and tumor segmentation on MRI in retinoblastoma patients; and Bouget et al. [67] used three-dimensional neural network architectures to automatically detect meningioma on MRI scans.

The AI algorithms have represented strengths in detecting tumor patterns by identifying underlying genetic and molecular characteristics associated with specific macroscopic tumor features based on medical images and/or high-throughput data. Zhao et al. [32] reported that ML-based radiomics algorithms could predict *H3 K27M* amplification status in children with midline glioma with significantly greater accuracy ranging from 0.788 to 0.867 than prediction by chance. Giwa et al. [68] applied ML-based algorithms in predicting N-myc status and survival risk using CpG methylation in children with neuroblastoma.

## Challenges and future outlook

The application of AI technology faces some important challenges that must be resolved to ensure its use in pediatric cancer diagnosis [69]. For example, medical imaging data cannot be used as input data directly. It is crucial to extract features from the imaging data and process them. Development and popularization of technology, in addition, the weights coefficient in CNN models are tested, calculated, and the confidence interval is reasonable, so medical interpretation needs further research [70]. While the importance of AI to this field is recognized, the joint efforts of computer experts and medical experts toward ensuring interdisciplinary personnel training and collaboration are crucial. Only then can the potential of this technology be put to a practical and economic application by medical staff [71]. The possibility is that the “black box” of ML/CNN applications will reduce physician skills and soon transform some sectors of healthcare in ways that may appear to be practical and economic but with unintended negative consequences. Another crucial issue with regard to the future of AI in medicine involves privacy and data security assurances [72]. While recent years have witnessed much enthusiasm about the potential of “big data” and ML-based solutions, to date, only a few examples exist to illustrate the impact of AI on current clinical practice [73, 74]. The stimulating debate that whether AI is “smarter” than human practitioners is largely irrelevant, and we will consistently improve our collective health by using every information and data resource [10].

## Conclusions

AI techniques have revolutionized the diagnostic field of oncology. Although AI approaches have been widely implemented in adult tumors, specialized applications of AI algorithms in childhood cancer are still limited probably attributed to the insufficient amounts of available data sets. There are limited opportunities to transfer well-trained CNN architectures built on adults into pediatric oncology few CNNs are directly generalizable from adults to children. Therefore, it’s warranted urgently to develop dedicated AI algorithms applied in pediatric oncology. Although the data sets of pediatric oncology are not large enough to perform standardized DL analysis in medical imaging [75], the integration of detailed segmentation and standardized augmentation techniques [76] is expected to achieve satisfactory performance proven by recent literature on brain tumors [77]. Combined with a considerable amount of pediatric cancer lesions, DL-CNNs yield reasonable and promising performance in the field of pediatric oncology considering its nature of pixel-level classification. Based on currently collected data, the radiological images of brain cancers have great potential for AI implementation, which might be validated first for the assistance of clinical decisions in the near future. Further improvement of pediatric oncology diagnosis requires sharing of source data and algorithms among multiple institutions for standardizations and cross-validation to benefit subsequent treatment and surveillance maximally.

## Abbreviations

AI: artificial intelligence
ALL: acute lymphoblastic leukemia
AML: acute myeloid leukemia
AUC: area under the curve
CAD: computer-aided diagnosis
CML: chronic myeloid leukemia
CNNs: convolutional neural networks
DL: deep learning
lncRNAs: long non-coding RNAs
ML: machine learning
MRI: magnetic resonance imaging
RF: random forest

## Declarations

### Availability of data and materials

The datasets used and/or analyzed during the current study are available from the corresponding author on reasonable request. Requests for access to these datasets should be directed to [Yuan Li, l13258389785@126.com].

### Copyright

© The Author(s) 2023.
